# Spiritual Care in Action for Oncology Patients in the uMgungundlovu and eThekwini Health Districts, KwaZulu-Natal, South Africa: A Qualitative Study

**DOI:** 10.1177/23779608231193798

**Published:** 2023-08-16

**Authors:** Vashni Sewkarran, Emelda Zandile Gumede

**Affiliations:** 1 56394 University of KwaZulu-Natal, Durban, South Africa; 2 School of Nursing and Public Health, Howard College Campus, 56394 University of KwaZulu-Natal, Durban, South Africa

**Keywords:** health care institution, spiritual care, oncology patients, spiritual activities, professional nurse

## Abstract

**Introduction:**

Globally the inclusion of spiritual activities has been well adopted into nursing care. Empirical work related to the inclusion of spiritual activities in nursing within the South African context particularly within the oncology setting is sparse. Being diagnosed with cancer can be unsettling because of the severity and vigorous treatment that one has to endure. Spiritual activities could be used as one of the coping strategies for cancer patients in times of spiritual distress. Strengthening spiritual care by including spiritual activities into daily nursing care could be beneficial to the patient and the institution.

**Objective:**

This study aimed to explore spiritual activities that could strengthen spiritual care in oncology nursing practice.

**Methods:**

The research study followed a phenomenological qualitative descriptive design. The study population consisted of professional nurses working in the oncology units for more than 6 months. Four health care institutions were purposively selected. Data was collected through individual in-depth interviews and field notes. Ten professional nurses were recruited at the point of data saturation. Data analysis followed inductively using content analysis enrolling steps from Elo and Kyngas.

**Results:**

The findings revealed the nurse's willingness to embrace spiritual care in action into their daily nursing care. Three subthemes: holding patient's hands, hospital policy for patient visits, and music therapy outline how patients are comforted by nurses.

**Conclusion:**

Nurses are integral in the provision of spiritual care for cancer patients from diagnosis up until the end of life. The essence of spiritual activities gives patients a distraction and an escape from their pain and predicament thus improving their quality of life. The findings provide policy makers with an indication that patients do not only need medical and physical care but spiritual care in their nursing care plan.

## Introduction

Cancer is seen as a major health problem and it is becoming increasingly difficult to ignore how spirituality and spiritual activities in practice can assist cancer patients improve their well-being (Chandramohan, 2013; Siegel et al., 2017). To date, there is limited research on the inclusion of spiritual activities in oncology nursing practice in South Africa, whilst international research shows that spiritual activities assist patients cope with the potential consequences of cancer (O’Callaghan et al., 2016; Sibya et al., 2017; Sisk & Fonteyn, 2015).

Spiritual care assists individuals to cope better when faced with diseases like cancer. Patients have an inner feeling and believe that they are being supported by a higher power during these difficult times, hence improving their quality of life (Ebrahimi et al., 2017; Leeson et al., 2015). Researchers have noted this notion and deem it necessary that nurses include spiritual activities in their daily nursing care (Maciel et al., 2018; Sibya et al., 2017).

Nurses form the majority of the healthcare workforce in every country, including South Africa (Mitema et al., 2019). They spend the most amount of time with their patients and are the central players in saving and improving the quality of many patients’ lives. For patients who wish to use spiritual activities, this study could promote a greater acceptance of their illness and boost their well-being. The inclusion of spiritual activities into the spiritual care of oncology patients during their journey could become invaluable, in both public and private hospitals offering oncology nursing care. Spiritual activities should be routinely incorporated into holistic nursing care, to deliver more effective spiritual care.

## Review of Literature

The increasing incidence of malignancy is a worldwide phenomenon. Cancer is a cause of death before 70 years in 91 of 122 countries, including the majority of Western and Northern countries (Bray et al., 2018). Africa continues to be afflicted but Sub-Saharan Africa is particularly affected; it is among the countries that are predicted to have an increase of about 85% by 2030 (Morhason-Bello et al., 2013). This is attributed to scientific literature indicating low cancer awareness of less than 40% and low uptake of cancer screening tests in less than 20% of the population (Morhason-Bello et al., 2013). In comparison to high-income countries, the survival rate of cancer patients in Africa, including South Africa is far worse.

When oncology patients undergo treatment, they are susceptible to pain, nausea, vomiting, and increased stress levels which affects their well-being resulting in spiritual distress. Selman et al. (2018) confirm that spiritual distress is common among patients with diseases like cancer and lack of care results in suffering, patients being unhappy about their care, and increased health costs. Spiritual activities could be used as one of the coping strategies throughout periods of emotional stress like after the initial diagnosis, during chemotherapy, radiation, oncology surgery, and after being referred for palliative care, among other traumatic circumstances. Nurses are expected to recognize spiritual distress in their patients and failure to do so will harm the oncology patient's well-being (Calderia et al., 2017).

In this vein international, studies have shown that the incorporation of spiritual interventions namely, music, prayer, guided imagery, and yoga into spiritual care assists patients cope better with the devastating effects of cancer thereby improving their quality of life (Satija & Bhatnagar, 2017). In Australia, O’Callaghan et al. (2016) conducted a meta-ethnographic study, by examining five qualitative studies, in which he explored the role of music in people affected by cancer. The findings indicated that listening to one's selection of music is helpful when patients are undergoing diagnostic procedures, treatment, and palliative care. Multiple studies have also flagged that yoga is becoming more and more accepted as a complementary approach to reducing serious cancer-related symptoms (Danhauer et al., 2019; Sisk and Fonteyn, 2015). Similarly, a study by Sibya et al. (2017) noted that professional nurses also considered using aromatherapy, relaxation techniques, therapeutic touch, meditation, and support groups as alternative therapies in nursing practice.

Spiritual interventions are perceived as an important strength in individuals to manage their life challenges, and for South Africans living with cancer, this can be a source of comfort and strength during the recovery treatment process. South Africa is a multicultural country, comprising four (4) major ethnic groups namely, Blacks, Coloureds, Indians/Asians, and Whites (Fetvadjiev et al., 2015). The level of care required by individuals diagnosed with cancer is complicated and multifaceted and requires different models of care based on the needs of the population (National Policy Framework and Strategy on Palliative Care, 2017–2022). Within the South African context, each province and district may adopt a model or combination of models that best suit their needs. Currently, there are three different local models of care; the Abundant Life project at Victoria Hospital; an integrated community palliative care model initiated at South Coast Hospice in KwaZulu-Natal, and the Gauteng Centre of Excellence for Palliative Care based at Chris Hani Baragwanath Academic Hospital (National Policy Framework and Strategy on Palliative Care, 2017–2022).

The current study adopts the integrated community palliative care model that caters for the physical, psychosocial, and spiritual care needs of patients, that can be managed by professional nurses and caregivers in the patient's home or healthcare facilities (Drenth et al., 2018). On the other hand, some patients may experience psychosocial distress that requires a more specialized level of health care intervention at a district or regional hospital whilst others may have ongoing complex needs which require ongoing specialist-level interventions, either at a regional or tertiary level of care (National Cancer Strategic Framework for South Africa, 2017–2022).

Spiritual care should be provided throughout the entire trajectory of nursing care but the inclusion of spiritual activities in oncology nursing practice has never been included in the South African Health Care System. Hence, the study aims to explore spiritual activities that could strengthen spiritual care in oncology nursing practice in the uMgungundlovu and eThekwini Health Districts, South Africa. It is hoped that this study will endeavor to encourage nurses to include spiritual activities that are universal to all patients, in their daily activities.

## Methods

### Design

This study adopted a phenomenological qualitative descriptive design to explore spiritual activities that could strengthen spiritual care in oncology nursing practice, thus improving the quality of patients’ lives. The proposed method was deemed suitable; as a phenomenological study, it explored the participants lived experiences of caring for oncology patients as it occurred in their daily working lives (Creswell & Plano Clark, 2018).

### Research Question

1. What are the spiritual activities that could strengthen spiritual care and improve the quality of oncology patients’ lives?

### Sample

Non-probability purposive sampling was used to recruit participants from selected sites in the uMgungundlovu and eThekwini Health Districts. Two participants were interviewed from a private hospital, offering specialized oncology care; three from a public-private partnership hospital, offering central and tertiary care; three from a public hospital, offering tertiary care, and two from a non-government organization (NGO) offering oncology and palliative care.

A total of ten professional nurses from different oncology nursing units in the selected healthcare facilities were interviewed. The sample size in this study was determined by data saturation. Participants contributed rich information that was relevant to the purpose of the study and had the experience of nursing oncology patients.

### Inclusion and Exclusion Criteria

The inclusion criteria required professional nurses to have a minimum of 6 months of working experience in oncology units. The exclusion criteria were professional nurses working in oncology units that did not have 6 months of working experience and those who declared unwillingness to be interviewed.

### Data Collection

After gatekeeping arrangements were sorted, data was collected on dates and times best suited to the participants and not interfering with their daily activities. All interviews were conducted in English.

Semi-structured interviews made use of a voice recorder; field notes and probing were conducted. Interviews were conducted over 6 months from January 2022 to June 2022. All interviews were recorded on an audio tape recorder with permission from the participants, ensuring the comprehensiveness of information. Interviews continued until data saturation was reached. To ensure credibility, prolonged engagement was achieved by conducting interviews that lasted between 40 and 60 min. All data was stored in a password-protected computer.

### Data Analysis

Data was transcribed verbatim and thereafter analysed using content analysis in an inductive way enrolling phases (Elo & Kyngas, 2007). To ensure the anonymity of the participants no personal details were captured instead each interview was coded. The main themes were aligned with the study objectives (Table 1).

**Table 1.** Phases of Data Analysis Enrolling Steps From Elo & Kyngas (2007).

Phases involved	Procedure to ensure data analysis
Phase 1	Preparation The researcher read and re-read the data from the transcribed interviews to get a feeling of what the text was talking about.
Phase 2	Organizing During this process, the researcher read line by line and paragraph by paragraph identifying words, sentences, or paragraphs related to each other. While reading the researcher made notes in the margins. Then, the researcher grouped all data with one central meaning into units and labelled them with codes. Regular meetings were scheduled with a supervisor who is well versed with qualitative methodology to ensure that all coding was thoroughly examined before finalization The codes were then joined together according to their relationships to form categories that were related and finally into themes. To ensure credibility the participants were asked to review their transcripts, categories and themes to confirm that it was a true representation of their descriptions. In addition, the researcher discussed and refined the themes with the supervisor until consensus was reached and finally approved.
Phase 3	Reporting Lastly, to ensure transferability, the researcher gave a detailed description of the analysis process and the results so that, the readers know exactly how the analysis was executed, thus increasing the reliability of the study.

### Ethical Considerations

Permission was granted by the KwaZulu-Natal Provincial Department of Health on December 3, 2021 (NHRD Ref: KZ_202111_020) as the study is to be conducted in health facilities in KwaZulu-Natal.

The study was approved by the Biomedical Research Ethics Committee of the University of KwaZulu-Natal (BREC / 00003395/2021) on December 8, 2021. All participants provided written informed consent before enrolment in the study. Information regarding the research study was fully explained in an information letter, which was given to the participants and was explained by the researcher. Anonymity was maintained as participants did not have to reveal their names during the interview. During data analysis no personal details of the participants were captured instead each interview was coded. This study was conducted in conformance with the Helsinki Declaration.

## Results

### Sample Characteristics

Nine out of ten professional nurses were female. This is in line with the SANC Annual Statistics for 2021 as nursing is a female-dominated profession (SANC Distribution Stats, 2021). At the end of 2021, there were 31,699 female professional nurses compared to 4108 male professional nurses in KZN.

### Research Question Results

This study explored spiritual activities that could contribute to improving the quality of oncology patients’ lives and strengthen spiritual care. Based on the participant's responses, the following theme was generated “spiritual care in action” and three subthemes: holding patient's hands, hospital policy for patient visits, and music therapy.

## Spiritual Care in Action

Patients bring their spiritual beliefs to the hospital and it is the nurse's responsibility to embrace them. As such this theme outlines how the patients are comforted by nurses during their daily activities.

### Holding a Patient's Hand

As healthcare providers, nurses play a critical role in providing holistic care to oncology patients by addressing their spiritual needs. Participants stated that they can provide spiritual care by showing kindness, listening to patients, allowing patients to explore their fears, and holding their hands during their daily nursing care. In doing so, nurses take cognizance of the patient's beliefs, values, and existential concerns. Participants stated that:

You stop and listen to them and provide reassurance. Sometimes you hold their hands [P10]

As an oncology nurse, you need to listen, hold their hands, counsel, empathize and walk this journey with your patients. Show compassion, understanding, be patient and humble [P6]

Nurses who are aware of patient's spiritual needs and preferences can provide compassionate care by holding patients hands so that patients can feel their presence and readiness to assist and support them, enhancing the patient's well-being

Most pastors when praying and singing, the patient's souls get lifted, let alone if they hold their hands, they offer comfort and compassion [P6].

Another participant clarified that patients are also comforted by significant others like pastors who provide comfort by holding their hands thus improving their psycho-social well-being.

We offer ourselves in the form of a smile, holding their hands while reading from their bible, nodding of our heads, just to give them that smoother transition into their terminal illnesses, especially those that are dying [P8]

Participants emphasized that even simple touch like holding their hands can make a patient's journey less daunting as they are not scared and alone during this difficult time. Reading to them from their sacred books will provide comfort and ease any spiritual pain experienced.

In most cases, mothers do not stay with their child. In her absence, we comfort the child by hugging them, holding their hands, and offering them toys to play with especially during procedures like injections, so that they don’t feel the pain or feel unloved and isolated [P5]

Besides adults, children also need nurturing and comfort during procedures to keep their minds off any discomfort and pain. Nurses are happy to embrace children and provide distractions so that the child receives the treatment modalities much needed for recovery.

When we do our home visits for terminally ill patients, we simply sit with them, hold their hands and speak to them calmly and reassuringly [P1]

Another participant stated that for terminally ill patients, home visits are beneficial. The patient might not be able to speak and respond to you, but they most probably can hear you and feel your touch allowing the patient to live his life to the fullest.

### Hospital Policy for Patient Visits

The family or significant others play a very pivotal role in the oncology patient's treatment journey. This subtheme finds that a modified hospital policy in line with critically ill patients does contribute to spiritual wellness as patients appreciate the time spent with their loved ones. Participants stated that:

We do have a visiting hours’ policy, which allows two family members per visiting hour [P4]

Visitors follow the hospital policy for patient visits, to allow a peaceful and quiet environment for patients to recuperate and to allow clinical staff to carry on with their duties. However, the patient needs to have visitors as patient and family engagement can help patients recover fast. Participants also expressed that:

For critically ill patients, we allow family members extended visiting hours, they can stay with the patient as long as they want, provided it does not interfere with patient care [P6]

The oncology nurse who has embraced her spirituality will use this privilege of the flexibility of visiting hours in the best interest of her patients by applying her discretion very well.

Our patients are from far and they do not get visitors often. They are anxious about their condition, families, children, and their jobs, therefore a visit by their priest during a suitable time will be beneficial [P5]

Besides family visits, priests were allowed to visit patients outside of the normal visiting hours and without interfering with the routine of the ward. Patients from far will often feel completely alone and isolated. Many patients do not have emotional support or anyone to uplift their spirits. Pastors can provide emotional spiritual guidance to speed up the healing process

We do make special consideration for patients who are critically ill and family who are from very far. We allow family extended visiting time with their loved ones to make positive memories and simply to be there with them [P6]

Usually, visitors need to adhere to the hospital policy for patient visits, but participants allow family extended visiting time with critically ill patients so that they can make memories and support their loved ones, improving the patient's well-being.

### Music Therapy

Music is a vital part of the treatment of cancer patients as it carries away their minds from the pain and is seen as an emotional soother. Participants emphasized that music improves the patient's moods, their quality of life and reduces isolation.

We believe that music relieves boredom and loneliness and helps patients to relax and gives them something else to focus on rather than the treatment process [ P10]

The participants felt that music therapy improves the patients coping skills, alleviates depression, and improves their well-being.

Generally, our patients are sad, in pain, and sometimes uncomfortable because of treatment. Listening to music will improve their mood, calm their minds and assist with the uptake of sedation [P6].

Due to the vigorous nature of the treatment, patients experience lots of pain. Participants emphasized that music therapy can alter the patient's perception of pain directing their attention away from the pain and making them comfortable.

Play and music are very beneficial because it takes the child's mind off their pain. They don’t even cry when we are giving them an injection, they are so occupied with this lady [ P5]

It is important to note that music therapy has an impact on the child's well-being. Participants reported that music therapy helps children manage pain and stressful situations.

Yes, they walk with a loudspeaker singing and praising God. There was a toddler, he was dancing while listening to the music, we were so impressed and they were laughing. It was so nice to see the children laughing and happy as most of the time they are very sad [P7]

Hospitalization for children can be very stressful. Participants believe that music therapy allows children to express themselves. Music allows them to express their emotions and moods making an unpleasant situation into a pleasant one.

## Discussion

Cancer treatment, particularly end-of-life care is by no means easy. This position is taken because in end-of-life care, patients start to wonder what the point of their lives is, and they feel anxious about death, the process of dying, and the consequences of these events (Calderia et al., 2017). As a result of this existential contemplation, a significant number of hospitalized patients do not have their spiritual needs addressed and do not receive adequate spiritual care, resulting in spiritual distress (Calderia et al., 2017). In this vein, international studies have shown that the incorporation of spiritual interventions namely, music, prayer, and guided imagery into spiritual care assists patients to cope better with the devastating effects of cancer thereby improving their quality of life (Satija, & Bhatnagar, 2017). The failure of nurses to recognize and address spiritual care is a challenge that should be tackled head-on (Calderia et al., 2017).

Professional nurses are expected to provide spiritual care to patients. However, Ross et al. (2022) argue that this is not the case as nurses feel inadequately prepared for it. To strengthen spiritual care and reduce patients’ stress levels, spiritual activities like holding a patient's hand, allowing unlimited visiting time for critically ill patients, and the inclusion of music therapy should be part of daily nursing practice. Kelly et al. (2021) agree that spiritual care like supporting patients, listening to and exploring their fears, and showing kindness while providing care assist in dealing with their issues.

### Holding a Patient's Hand

Due to the nurse's busy schedules, it is well understood that it becomes increasingly difficult for them to embrace spiritual care in their daily activities. Sandnes and Uhrenfeldt (2022) confirm that dealing with critically ill patients can be very challenging over time. Likewise, nursing patients with cancer is no different from nursing critical patients with any other diagnosis. Oncology patients are always facing a crisis or are in spiritual distress due to the nature of their illness. Nurses must be empowered to appreciate patients’ different religions and beliefs to embrace spiritual care in action.

In this study, although participants experience busy days, they agreed that a little contact with patients like holding their hands during their daily activities like administering medication or bathing can be of great comfort to patients. In doing so, professional nurses display a caring, empathetic, and dignifying side of their nursing care. This may be only what patients need to take them through that difficult journey. Holding their patient's hand is characteristic of physical touch and caring which may have a positive effect on the patient's well-being and recovery. Travelbee Human-to-Human Relationship model (1971) confirms that the main goal of the nurse is to assist the patient cope with their illness, lessen and/or alleviate the patient's suffering, and restore their health thus improving their quality of life (Parola et al., 2020).

The act of holding a patient's hand during daily nursing activities is centered around compassion, promoting comfort, promoting emotional comfort, promoting mind and body comfort, promoting a social role, and sharing spirituality, which is in line with the objective of the study (Sandnes and Uhrenfeldt, 2022).

### Hospital Policy for Patient Visits

A hospital policy for patient visits is very crucial for meeting patients’ spiritual needs. The participants concur that both friends and families play a significant role in mitigating spiritual distress. Traditionally, patient visits were restricted for various reasons like maintaining order as visitors can be disruptive and noisy and preventing the spread of infection (Belanger et al., 2017). However, participants in the study agreed that a modified hospital policy in line with critically ill patients will contribute to spiritual wellness as patients appreciate the time spent with their loved ones, embracing spiritual care in action.

Belanger et al. (2017) confirm that healthcare facilities do recommend adopting an open visiting hours’ policy. The reason for this is related to the fact that healthcare facilities have an end goal, intending to have both the patient and family as partners involved in care.

Furthermore, participants stated that nurses are allowed to call patients’ priests to come to visit anytime to offer spiritual care to oncology patients, embracing spiritual care in action. This process gives the patients the encouragement they need; the feeling of forgiveness, and the need to be given a greater hope that they will one day be free of pain in a spiritual dimension. The major finding is that the institutions are in collaboration with the priests and families of critically ill patients to promote peaceful death. This notion is supported by Ebrahimi et al. (2017) who agreed that spiritual care including spiritual activities assists individuals to cope better when faced with diseases. It promotes inner feelings and the belief that they are being supported by a higher power during these difficult times, hence improving their quality of life.

### Music Therapy

The major finding from this subtheme is that music is an effective alternative therapy that can be used in conjunction with spiritual care to comfort and support patients undergoing cancer treatment. Nightingale (1859) as quoted by Dossey (2010) believed that using music as a form of diversional or alternate therapy like singing and using musical instruments are of great benefit to patients. Similarly, in Australia, O’Callaghan et al. (2016) reported that music has a beautiful, aesthetic experience on cancer patients of all ages, including their caregivers. It can be more effective than language when communicating feelings and emotions. Overall music can offer hope for survival, endure stress, encourage a sense of well-being, and eases loneliness (O’Callaghan et al., 2016).

In daily nursing practice, the following can be made available to patients who wish to use music; musical instruments, recordings, and suggesting that patients bring their personalized music into the hospital. On the other hand, patients might associate specific music with feelings of stress and loss that were encountered during their treatment or procedures. Therefore, O’Callaghan et al. (2016) emphasizes that for patients who do not want to listen to music during their illness, their desire for avoiding music must be respected by nurses.

Another crucial finding is that these spiritual activities also differ according to the age of the patients, as children too can identify with music and play therapy in their environment. This notion is supported by O’Callaghan et al. (2016) who agree that music is found to be symbolic, in that children can relate to musical play in their environment. Some children have been found to want play as it soothes the pain away. Therefore, healthcare professionals need to be aware of their role in extending the value of music and how to offer music-based support to cancer patients (O’Callaghan et al., 2016).

### Strengths and Limitations

One hospital delayed the gatekeeper approval for 3 months, thus delaying the data collection process. Furthermore, this study was restricted to professional nurses in oncology units only, therefore future research would benefit from expanding this research to other health professionals and units like medical and surgical units. However, since the participants provided rich information is an indication that this strength will outweigh the limitation and include spiritual activities across all disciplines in nursing. The participants in the study had more than 6 months’ experience with the majority having between 10 and 20 years of experience characterized by extensive years of nursing knowledge and clinical exposure. This confirms that the participants are not a novice but instead, they are skilled nurses as they have had an opportunity to put what they have learned in the classroom into practice.

### Implications for Practice

It is believed that through the introduction of spiritual activities in oncology nursing practice, professional nurses would adopt a spiritual paradigm in their nursing practice. It would equip professional nurses with the knowledge to intervene in this area daily by assisting patients who consider using spiritual activities to improve their quality of life during their illness. It is envisaged that this study will create awareness of the importance of spiritual activities in oncology practice.

Furthermore, managers of institutions would also become more aware of the importance of this field, thus possibly leading to introducing spiritual activities across all other disciplines like medical and surgical units. This study should allow managers and professional nurses to realize that patients do not only need medical and physical care but spiritual care in the nursing care plan.

## Conclusion

The study concluded that the essence of these spiritual activities is to give oncology patients a distraction and to give them an escape from their pain and predicament, therefore complementing spiritual care.

It is recommended that a module on effective spiritual care protocols be included in the new post-basic oncology program, in-service training, and workshops to boost the nurse's levels of confidence in spiritual care. Furthermore, this study was restricted to professional nurses only, therefore future research would benefit from expanding this research to other healthcare professionals, as oncology care is a team approach.
